# The impact of the end of COVID confinement on pandemic dreams, as assessed by a weekly sleep diary: a longitudinal investigation in Italy

**DOI:** 10.1111/jsr.13429

**Published:** 2021-07-20

**Authors:** Serena Scarpelli, Maurizio Gorgoni, Valentina Alfonsi, Ludovica Annarumma, Valentina Di Natale, Emilio Pezza, Luigi De Gennaro

**Affiliations:** ^1^ Department of Psychology Sapienza University of Rome Rome Italy; ^2^ IRCCS Fondazione Santa Lucia Rome Italy

**Keywords:** COVID‐19, dreaming, Italian lockdown, lucid dream, pandemic, sleep

## Abstract

The Coronavirus 2019 pandemic strongly affected our sleep and dream activity. Many cross‐sectional studies highlighted increased dream recall frequency, and revealed a great presence of pandemic‐related oneiric contents. Here, we present the first prospective study carried out on an Italian sample. One‐hundred subjects were requested to fill out a web‐survey including socio‐demographic information, and questionnaires collecting sleep and clinical measures during lockdown. A final sample of 90 subjects participated in the longitudinal protocol lasting 2 weeks: (a) the first week (April 28–May 4) of full lockdown; and (b) the second week (May 5–May 11) of easing of restrictions. Subjects were asked to record at home their dream experiences, and complete a sleep‐dream diary each morning. Statistical comparisons showed that participants had higher numbers of awakenings, lower ease of falling asleep, higher dream recall and lucid dream frequency during lockdown than post‐lockdown. Further, subjects reported more dreams, including “being in crowded places” during post‐lockdown than lockdown. The poorer sleep quality during lockdown is quite consistent with previous findings. The relationship between traumatic events and dream recall frequency confirmed the idea of pandemic as “collective trauma”. Also, we hypothesized that the greater presence of lucid dreams during confinement could reflect the attempt to cope with the waking pandemic‐experiences. Finally, the presence of crowded places into dream scenarios during the second week of our protocol appears consistent with the continuity‐hypothesis, as the possibility to access places frequented by other people could represent a relevant experience after a long period of confinement.

## 1 INTRODUCTION

The Coronavirus 2019 (COVID‐19) outbreak critically impacted the worldwide population. In the first phase of the pandemic, Italy was one of the most affected countries, and the government quickly adopted restrictive measures between March and May 2020 to contain the infection. Confinement and social distancing provoked remarkable daily life changes that significantly influenced sleep patterns (Blume et al., 2020; Casagrande et al., 2020; Cellini et al., 2020; Franceschini et al., 2020; Wright et al., 2020) and oneiric activity (Gorgoni et al., 2021; Iorio et al., 2020; MacKay & DeCicco, 2020; Mota et al., 2020; Pesonen et al., 2020; Scarpelli, Alfonsi, Mangiaruga, et al., 2021; Schredl & Bulkeley, 2020).

Cross‐sectional web‐surveys carried out among Italian people revealed that more than half of participants reported poor sleep quality (Alfonsi et al., 2021; Casagrande et al., 2020; Franceschini et al., 2020), and sleep timing changes were found in parallel with sleep difficulties (Cellini et al., 2020). In addition, increased sleep disturbances and hypnotic medications were observed (Jahrami et al., 2021). Also, sleep complaints were related to psychiatric symptoms, such as anxiety and mood alterations (Casagrande et al., 2020; Cellini et al., 2020; Franceschini et al., 2020).

In parallel, sleep patterns have been proven to affect dreaming during lockdown (Scarpelli, Alfonsi, Gorgoni, et al., 2021), and poor sleepers reported qualitatively richer dreams than good sleepers (Gorgoni et al., 2021).

A cross‐sectional investigation in the USA showed higher dream recall frequency (DRF), negative emotions, and pandemic‐related dream contents (Schredl & Bulkeley, 2020). Consistently, Pesonen et al. (2020) revealed pandemic‐related contents in dreams linked to waking distressing events. Further, MacKay and DeCicco (2020) found that the dream scenario during the Canadian COVID‐19 experience was characterized by several contents typically related to daytime anxiety. An investigation among Chinese people revealed a higher frequency of frightening pandemic‐related dreams, linked with significant psychological distress (Wang et al., 2020).

It should be noted that some stable demographic features impacted dream activity during lockdown, such as age and gender. Italian studies revealed that women had a higher dream recall rate and negative emotions than men (Gorgoni et al., 2021; Iorio et al., 2020). Barrett (2020) confirmed this gender effect by comparing pandemic dream reports with normative dreams. Further, Scarpelli, Alfonsi, Mangiaruga, et al. (2021) highlighted that aging affected dream recall and nightmare frequency also during lockdown.

A limited number of studies tried to detect dreaming changes comparing oneiric activity during confinement with self‐ratings referred to pre‐pandemic period or normative values. Specifically, Scarpelli, Alfonsi, Mangiaruga, et al. (2021) compared a large Italian sample with a population‐based sample collected in 2019, showing increased dream recall and nightmare rates during the Italian lockdown (Scarpelli, Alfonsi, Gorgoni, et al., 2021). Gorgoni et al. (2021) asked subjects to report, retrospectively, information on their dream activity before the lockdown, finding higher DRF, negative emotions, bizarreness, vividness and dream length than pre‐pandemic periods. Furthermore, Mota et al. (2020) compared pandemic dreams with a set of dreams collected before the pandemic. The authors revealed a greater presence of negative emotions and pandemic‐related dream contents than the pre‐pandemic period (Mota et al., 2020). However, in this study some participants provided data only pre‐pandemic, and some only during the pandemic, so it could be describe as mainly cross‐sectional design.

Overall, these findings point out that pandemic, and especially lockdown, altered dream activity both from a qualitative and quantitative perspective. Dream features were frequently associated with personal concerns of our waking state and emotional processes, as highlighted by the “continuity‐hypothesis” (Scarpelli, Alfonsi, Gorgoni, et al., 2021).

However, the cross‐sectional nature of most of the above‐mentioned studies represents a strong limitation, and only Mota et al. (2020) provide analyses on dream reports longitudinally collected during the pandemic.

As compared with the prospective‐longitudinal method, the retrospective questionnaires allowed researchers to quickly collect data from large samples and with low expenses. On the other hand, this method is prone to considerable memory biases, leading to underestimating the dream recall rate (Zadra & Robert, 2012). The cross‐sectional protocol allowed only between‐subjects design, and ruled out to detect and control for basic inter‐individual (trait‐like) differences in dream activity.

Here, we present the first within‐subjects longitudinal study carried out on dream activity in an Italian sample across the pandemic. We used a prospective method (daily home logs and systematic dream recordings) to overcome the underestimation problem and mnestic biases (Zadra & Robert, 2012). The great advantage consists in the possibility to monitor intra‐individual changes in dream experiences over time (Zadra & Robert, 2012). Specifically, we aimed to identify modifications in qualitative and quantitative dream features between lockdown and the first week after the ease of restrictions (post‐lockdown). Consistently with the current literature, we expected to find higher DRF and qualitative features (e.g. emotional intensity, vividness) during lockdown than post‐lockdown. Also, we hypothesized to find fragmented sleep during lockdown compared with the post‐lockdown.

## 2 METHODS

### 2.1 Subjects and study design

One‐hundred subjects were recruited through social media (Facebook, Instagram, Whatsapp) during the Italian lockdown to participate in the study. Firstly, they were requested to fill out a web‐survey including socio‐demographic information, and self‐administered questionnaires to collect sleep and clinical measures during lockdown.

Then, subjects were trained to participate in the longitudinal protocol for 14 days, from April 28 to May 11, 2020. Specifically, the first week (April 28–May 4) was the last period of full lockdown, and the second week (May 5–May 11) represented the first period of easing of restrictions. They were asked to record at home their dream experiences each morning by dictating them into a handheld tape‐recorder, and fill out a sleep and dream diary. Participants were instructed to record their dreams within 15 min after awakening. Furthermore, they were recommended to give as accurate description as possible of all aspects of the dream experienced and, when more than one dream was recalled, to specify when a distinct dream was going to be reported. Audio recording was chosen as it provides a more accurate report of dream mentation and a higher compliance than a written report (Casagrande & Cortini, 2008).

After dream tape‐recording, a sleep‐dream diary was filled out in order to collect subjective estimates of the characteristics of the overall night‐time sleep and the self‐reported quantitative and qualitative dream features. The procedure was repeated identically for both weeks.

Each participant signed the informed consent form, declaring: (a) the explicit agreement to participate in the research; and (b) age ≥ 18 years. At any moment, the participant could withdraw from the protocol. No monetary compensation was provided for the participation. The study was approved by the Institutional Review Board of the Department of Psychology of the Sapienza University of Rome (#0000646/2020), and was conducted in accordance with the Declaration of Helsinki.

The final sample included 90 subjects (age range: 19–41 years; mean ± standard deviation: 25.77 ± 3.85) as 10 participants withdrew from the study before the 2 weeks provided for the protocol. Socio‐demographic features of the sample were reported in Table 1.

**TABLE 1:** Socio‐demographic features of the final sample

Final sample *N* = 90		
	*N*	%
Gender		
Female	72	80
Male	18	20
Italian area		
North	6	6.7
Centre	36	40
South	48	53.3
Education		
High school	37	41.1
Undergraduate	30	33.3
Graduate	14	15.6
Post‐graduate	9	10
Occupation		
Employed	31	34.4
Student	50	55.6
Unemployed	9	10
Cohabitation during lockdown		
Family/partner	80	88.9
With cohabitants	7	7.8
Living alone	3	3.3

### 2.2 Instruments

#### 2.2.1 Web‐survey

##### Demographic information

An online questionnaire was administered to evaluate demographic data (i.e. age, gender, education, occupation).

##### Anxiety symptoms

The State‐Trait Anxiety Inventory (STAI‐Y, I‐II; Spielberger, 2010) was administered to evaluate anxiety symptoms. It is a self‐reported questionnaire, consisting of 40 items: 20 for the STAI‐Y I (state‐like anxiety assessment); and 20 for the STAI‐Y II (trait‐like anxiety assessment). Subjects was required to rate how they feel (“at the moment of the administration” for STAI‐Y I, or “generally” for STAI‐Y II) on a 4‐point Likert scale from nothing to very much (cut‐off: scores ≥ 40 indicate clinically relevant anxiety symptoms).

##### Depressive symptoms

The Beck Depression Inventory‐II (BDI‐II; Beck et al., 1996) is a self‐administered questionnaire to assess depressive symptoms using 21 multiple‐choice questions. Each answer has a 4‐point scale that indicates degree of severity (from 0 = not at all to 3 = extreme form of each symptom). Total score > 13 is indicative of a clinically relevant depressive disorder.

##### Sleep quality and post‐traumatic stress disorder (PTSD) measures

The Pittsburgh Sleep Quality Index (PSQI; Curcio et al., 2013) is a self‐reported questionnaire used to assess the sleep quality during the last month (i.e. during the lockdown period) containing 19 items. The items provided information about self‐reported sleep quality (C1), sleep latency (C2), sleep duration (C3), habitual sleep efficiency (C4), sleep disturbances (C5), use of sleep medications (C6), and daytime dysfunction (C7). A PSQI global score > 5 indicates a subjectively perceived poor sleep quality. Further, in order to evaluate the presence of trauma‐related subjective sleep disturbances, we also required participants to fill out the PSQI‐Addendum (PSQI‐A; Germain et al., 2005). The PSQI‐A allows to assess seven disruptive nocturnal behaviours related to PTSD: flashes; general nervousness; memories or nightmares of traumatic experience; severe anxiety or panic not related to traumatic memories; bad dreams not related to traumatic memories; episodes of terror or screaming during sleep without fully awakening; episodes of acting out dreams, such as kicking, punching, running or screaming (cut‐off: score ≥ 4 is predictive for discriminating between subjects with and without PTSD).

#### 2.2.2 Sleep and dream diary

After dream tape‐recordings, the sleep‐dream diary was filled out by participants to collect information about sleep patterns and self‐reported dream qualitative and quantitative features (De Gennaro et al., 2011; Polini et al., 2017). Specifically, the following information on sleep was required: bedtime; sleep latency; ease of falling asleep (6‐point Likert scale from very difficult to very easy); number and duration of awakenings across the night; wake‐up time. Concerning dream features, the questionnaire required: the number of dreams recalled (dream frequency); the number of lucid dreams recalled (lucid dream frequency, i.e. the experience of being aware of dreaming during sleep); the emotional intensity; the visual vividness; the bizarreness and the perceived length of dream contents. Participants assessed each of these qualitative features by a 6‐point Likert scale.

### 2.3 Data analysis

#### 2.3.1 Sleep measures

Sleep measures obtained from sleep diaries were individually averaged as a function of the number of days (7 days) both at T0 (lockdown) and T1 (post‐lockdown). The dependent variables were: (1) sleep latency (min); (2) ease of falling asleep; (3) total sleep time (TST; min); (4) total bed time (TBT, min); (5) number of awakenings; (6) time spent in wake after sleep onset (WASO; min); (7) sleep efficiency index (SEI: TST/TBT%).

#### 2.3.2 Dream measures

The dream recordings were transcribed verbatim into overall daily reports. An expert researcher preliminarily pruned the report of each dream of the sentences and clauses not directly related to dream contents (e.g. “I'm not sure, but I think”) or contents repetition (De Gennaro et al., 2011; Polini et al., 2017; Scarpelli, D'Atri, et al., 2020). Then, two experts, unaware of the study protocol, assigned distinct scores to each dream report independently, according to three 6‐point Likert scales (from very small extent to very great extent) of emotional intensity (emotional load, EL), visual vividness (VV) and bizarreness (B). Further, two different scores were provided for positive (E+) and negative (E−) emotions (scores from 1 = low E+ or E− to 3 = high E+ or E−).

Regarding VV, the two judges assigned a score following these criteria: (1) no image at all (only thinking of the object); (2) very vague and dim; (3) less vague, still dim; (4) moderately clear and vivid; (5) clear and reasonably vivid; (6) perfectly clear and vivid as normal vision. The B score was assigned considering both bizarre elements (improbable or impossible characters, metamorphoses, improbable or impossible actions/inappropriate roles, improbable or impossible objects) and script bizarreness (physically improbable or impossible plot, logically improbable or impossible plot, plot discontinuity, improbable or impossible settings).

Finally, dream contents were categorized by the judges based on an adaptation of the *Typical Dream Questionnaires* (TDQ; Nielsen, 2012). The contents were illustrated in Table 2.

**TABLE 2:** Categorization of dream contents

Dream contents
1. Being chased or pursued, but not physically injured
2. Being injured
3. Being physically attacked (beaten, stabbed, raped, etc.)
4. Trying again and again to do something
5. Being frozen with fright
6. Food, eating
7. Arriving too late, e.g. missing a train
8. Swimming
9. Being isolated/locked up/shut down
10. Pets
11. Money
12. Flying or soaring through the air
13. Falling or being on the verge of falling
14. Being inappropriately dressed
15. Being nude
16. Being tied, unable to move
17. Being infected by a virus
18. Having superior knowledge, superpowers or magic abilities
19. Seeing him/herself in the mirror
20. Natural disasters (earthquakes, floods, tornados, …)
21. Insects, spiders or snakes
22. Being a member of the opposite sex
23. Being an object (e.g. tree or rock)
24. Encountering a kind of evil force, monsters or demon
25. Your teeth falling out/losing your teeth
26. Being killed or seeing yourself as dead
27. Vividly sensing, but not necessarily seeing or hearing, a presence in the room
28. Being unable to find, or embarrassed about using a toilet
29. School, teachers, studying
30. Sexual experiences
31. Losing control of a vehicle
32. Fire
33. A person now dead as alive
34. A person now alive as dead
35. Failing an examination
36. Suffocation, breathing problems
37. Feral and violent animal
38. Pandemic/epidemic
39. Being at a movie/cartoon/videogame/comic book
40. Killing someone
41. Lunatics or insane
42. Being half awake and paralysed in bed
43. Seeing a face very close to you
44. Seeing a UFO or an extra‐terrestrial
45. Being an animal
46. Being a child again
47. Seeing an angel or encountering God in some form
48. Discovering a new room at home
49. Airplane crash
50. Someone having an abortion
51. Being sick
52. Being close to someone sick
53. Zombies
54. Dictatorship
55. Being betrayed
56. Being at the workplace
57. Loved ones (family, friends)
58. Being in crowded places (restaurants, clubs, concerts, …)
59. War
60. Travelling
61. Social media interactions (video calls, chats, …)
62. Be possessed

The two judges were preliminarily trained on the basis of the database of dream report scores used in previous studies (Polini et al., 2017; Scarpelli, D'Atri, et al., 2020).

The dream reports of the current study were blindly scored with respect to the condition belonging (T0 or T1). Also, the inter‐rater reliability for each rating scale was very high (K > 85). Discordances between the two judges were consensually solved. Both the dream recall rate and lucid dream recall rate were computed as the average number of dreams reported by each subject per night, namely by dividing the total number of recalled dreams per 7 days. Moreover, the mean length of dream report per night was computed by dividing the total number of words of all the pruned reports by the number of days (7 or less) where subjects were capable of recalling one or more dreams (total word count; TWC) (De Gennaro et al., 2011; Polini et al., 2017). Also, the mean ratings of the qualitative characteristics (perceptual, emotional features and contents) of dreams were individually averaged as a function of the number of days where subjects were capable of recalling one or more dreams. Hence, the dependent variables were DRF, lucid dream frequency, self‐reported (from diaries) emotional load (sr_EL), self‐reported visual vividness (sr_VV), self‐reported bizarreness (sr_B), self‐reported length (sr_L), TWC, EL, VV, B, E+, E− and categorized dream contents (Table 2), calculated both at T0 (Lockdown) and T1 (Post‐Lockdown).

#### 2.3.3 Statistical analysis

All statistical analyses were performed using *Statistical Package for Social Sciences* (SPSS) version 25.0 and Matlab R2019.

Firstly, we performed a one‐way repeated‐measures multivariate analysis of variance (MANOVA), with “Time” (T0, lockdown versus T1, post‐lockdown) as within‐subjects factor, and sleep measures and dream frequency measures (traditional dream and lucid dream frequency) as dependent variables. In order to understand which conditions are different at the univariate level, we carried out univariate ANOVAs.

Then, considering the subgroup of participants that recalled at least one dream for the week both during T0 and T1, one‐way repeated‐measures univariate analyses of variance (ANOVA) were performed on each dream variable (sr_EL, sr_VV, sr_B, sr_L, TWC, EL, VV, B, E+, E−, and dream contents) between Lockdown and Post‐Lockdown to assess pandemic‐related changes in dream phenomenology.

For each analysis, *p*‐values < 0.05 were considered statistically significant.

## 3 RESULTS

### 3.1 Clinical features during lockdown

Sleep and clinical features retrospectively collected concerning the lockdown period are shown in Table 3. This information was available for 89 subjects as one subject did not complete the whole questionnaire. Among all respondents, 46.07% showed low sleep quality during lockdown, and 62.92% reported PTSD‐related symptoms during sleep. Further, 71.91% of the sample reported a significant level of state‐like anxiety, and 69.66% had a clinically relevant level of trait‐like anxiety symptoms. Finally, 25.84% of participants showed clinically relevant scores at BDI‐II.

**TABLE 3:** Sleep and clinical measures during lockdown obtained by web‐survey

	Mean ± SD	*N* 89; %	
PSQI global		PSQI ≤ 5	PSQI > 5
	6.17 ± 3.05	48; 53.93	41; 46.07
PSQI‐A		PSQI‐A ≤ 3	PSQI‐A > 3
	5.06 ± 3.36	33; 37.08	56; 62.92
STAI‐I		STAI‐I ≤ 39	STAI‐I > 39
	46.8 ± 10.91	25; 28.09	64; 71.91
STAI‐II		STAI‐II ≤ 39	STAI‐II > 39
	44.36 ± 10.00	27; 30.34	62; 69.66
BDI‐II		BDI ≤ 13	BDI > 13
	10.06 ± 7.00	66; 74.16	23; 25.84

Abbreviations: BDI‐II, Beck Depression Inventory – II; PSQI, Pittsburgh Sleep Quality Index; PSQI‐A, Pittsburgh Sleep Quality Index ‐ Addendum; STAI‐I, State‐Trait Anxiety Inventory ‐ I; STAI‐II, State‐Trait Anxiety Inventory ‐ II.

### 3.2 Changes in sleep patterns and dream frequency between lockdown and post‐lockdown

Among the total sample, 80 subjects reported at least one dream (min–max: 0–2.5 dreams per night) during the lockdown week (T0), while 10 subjects did not report any dreams. During the post‐lockdown week (T1), only 59 subjects reported at least one dream (min–max: 0–1.6 dreams per night).

**FIGURE 1:**
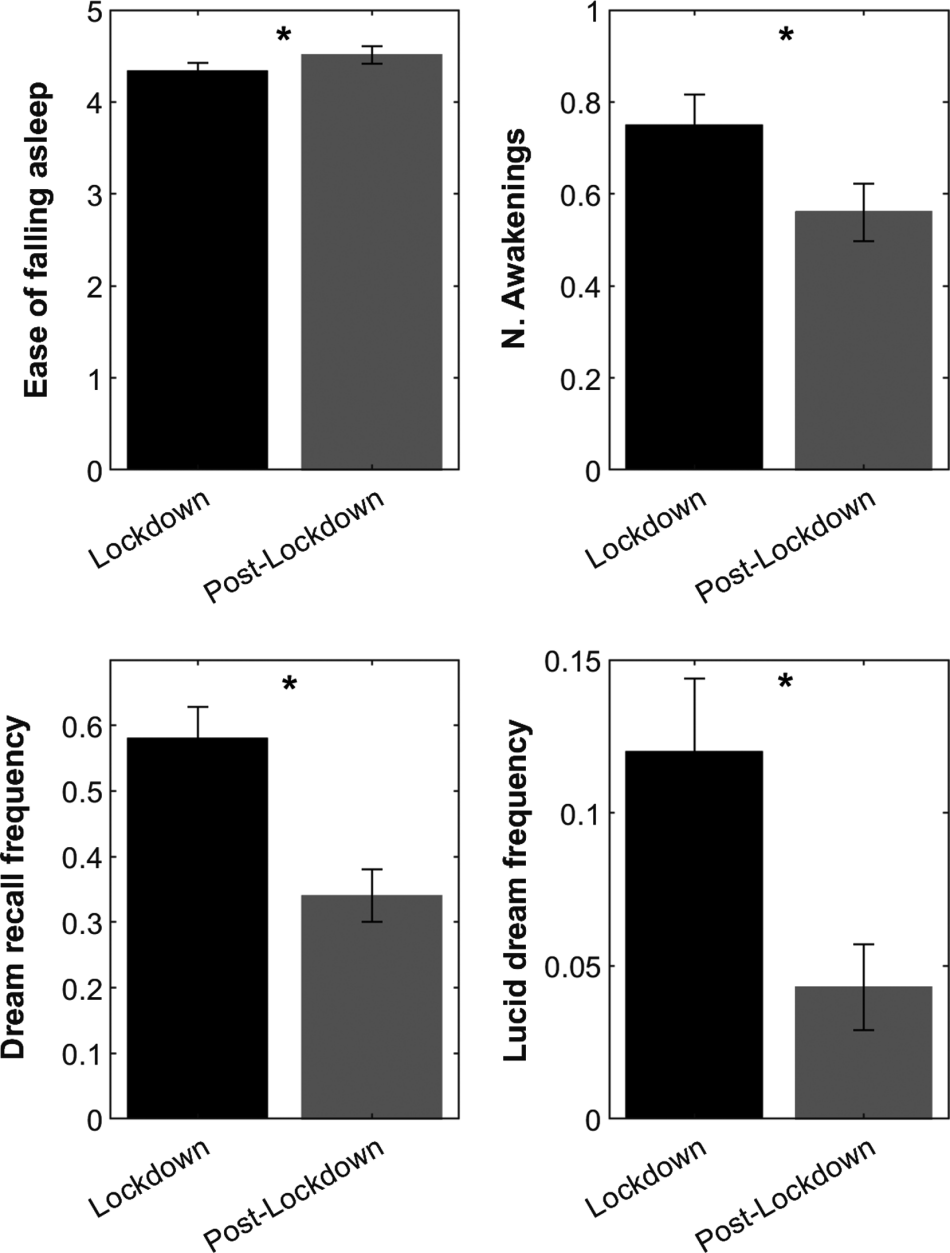
Sleep measures and dream frequency: comparisons between lockdown and post‐lockdown period. Significant results (asterisked; *p* < 0.05) of comparisons (MANOVA) between lockdown (black bars) versus post‐lockdown (grey bars) performed on sleep measures, dream recall and lucid dream frequency. Error bars represent the standard errors

One‐way MANOVA comparing the sleep patterns and dream frequency measures between lockdown and post‐lockdown showed a statistically significant difference (Wilks' *λ* = 0.555, *F*_9,81_ = 3.406, *p* < 0.001). Univariate ANOVAs revealed that subjects had lower ease of falling asleep (*F*_1,89_ = 4.47, *p* = 0.037) and higher numbers of awakenings (*F*_1,89_ = 16.67, *p* < 0.001) during lockdown than post‐lockdown. Further, DRF (*F*_1,89_ = 30.21, *p* < 0.001) and lucid dream frequency (*F*_1,89_ = 16.78, *p* < 0.001) were significantly higher during lockdown than post‐lockdown. Significant differences are shown in Figure 1. All results from univariate ANOVAs are reported in Table 4.

**TABLE 4:** Univariate ANOVAs (Lockdown versus Post‐lockdown)

	Mean (SE) Lockdown	Mean (SE) Post‐lockdown	*F*‐values (*p*‐values)	*η* ^2^
SOL	21.15 (1.79)	18.56 (1.79)	2.96 (0.089)	0.032
Ease of falling asleep	4.33 (0.09)	4.51 (0.1)	**4.47 (0.037)**	**0.048**
TST	461.78 (4.66)	463.34 (5.34)	0.15 (0.70)	0.002
TBT	550.33 (5.97)	549.07 (6.29)	0.062 (0.80)	0.001
Number of awakenings	0.75 (0.07)	0.56 (0.07)	**16.68 (*p* < 0.001)**	**0.158**
WASO	6.54 (0.84)	7.94 (1.05)	1.57 (0.21)	0.017
SEI (%)	84.35 (0.79)	85.12 (0.85)	2.09 (0.15)	0.023
DRF	0.57 (0.05)	0.34 (0.04)	**30.21 (*p* < 0.001)**	**0.253**
Lucid dream frequency	0.12 (0.02)	0.04 (0.01)	**16.78 (*p* < 0.001)**	**0.159**

Values in bold indicate significant difference.

Abbreviations: DRF, dream recall frequency; SEI, sleep efficiency index; SOL, sleep‐onset latency; TBT, total bed time; TST, total sleep time; WASO, wakefulness after sleep onset.

### 3.3 Changes in qualitative features and contents of dreaming

Univariate ANOVAs (Lockdown versus Post‐lockdown), reported in Table 5, showed no statistically significant differences concerning dreaming's qualitative features.

**TABLE 5:** Univariate ANOVAs on dream qualitative features (Lockdown versus Post‐lockdown)

	Mean (SE) Lockdown	Mean (SE) Post‐lockdown	*F*‐values (*p*‐values)	*η* ^2^
sr_EL	3.59 (1.52)	3.72 (1.45)	0.628 (0.431)	0.011
sr_VV	3.35 (0.11)	3.57 (0.14)	3.304 (0.074)	0.054
sr_B	3.45 (0.13)	3.51 (0.15)	0.104 (0.748)	0.002
sr_L	2.97 (0.13)	3.08 (0.15)	0.702 (0.406)	0.012
TWC	154.43 (15.52)	184.16 (22.68)	2.101 (0.153)	0.035
EL	2.73 (0.15)	2.95 (0.17)	1.474 (0.230)	0.025
VV	3.35 (0.10)	3.46 (0.12)	0.833 (0.365)	0.014
B	2.42 (0.11)	2.34 (0.12)	0.350 (0.556)	0.006
E+	0.25 (0.05)	0.32 (0.06)	0.805 (0.373)	0.014
E−	0.68 (0.08)	0.79 (0.09)	0.963 (0.331)	0.016

Note

The analyses were limited to the subsample of participants that recalled at least one dream per week both during T0 and T1.

Abbreviations: B, bizarreness; E−, negative emotions; E+, positive emotions; EL, emotional load; sr_B, self‐reported bizarreness; sr_EL, self‐reported emotional load; sr_L, self‐reported length; sr_VV, self‐reported visual vividness; TWC, total word count; VV, visual vividness.

**FIGURE 2:**
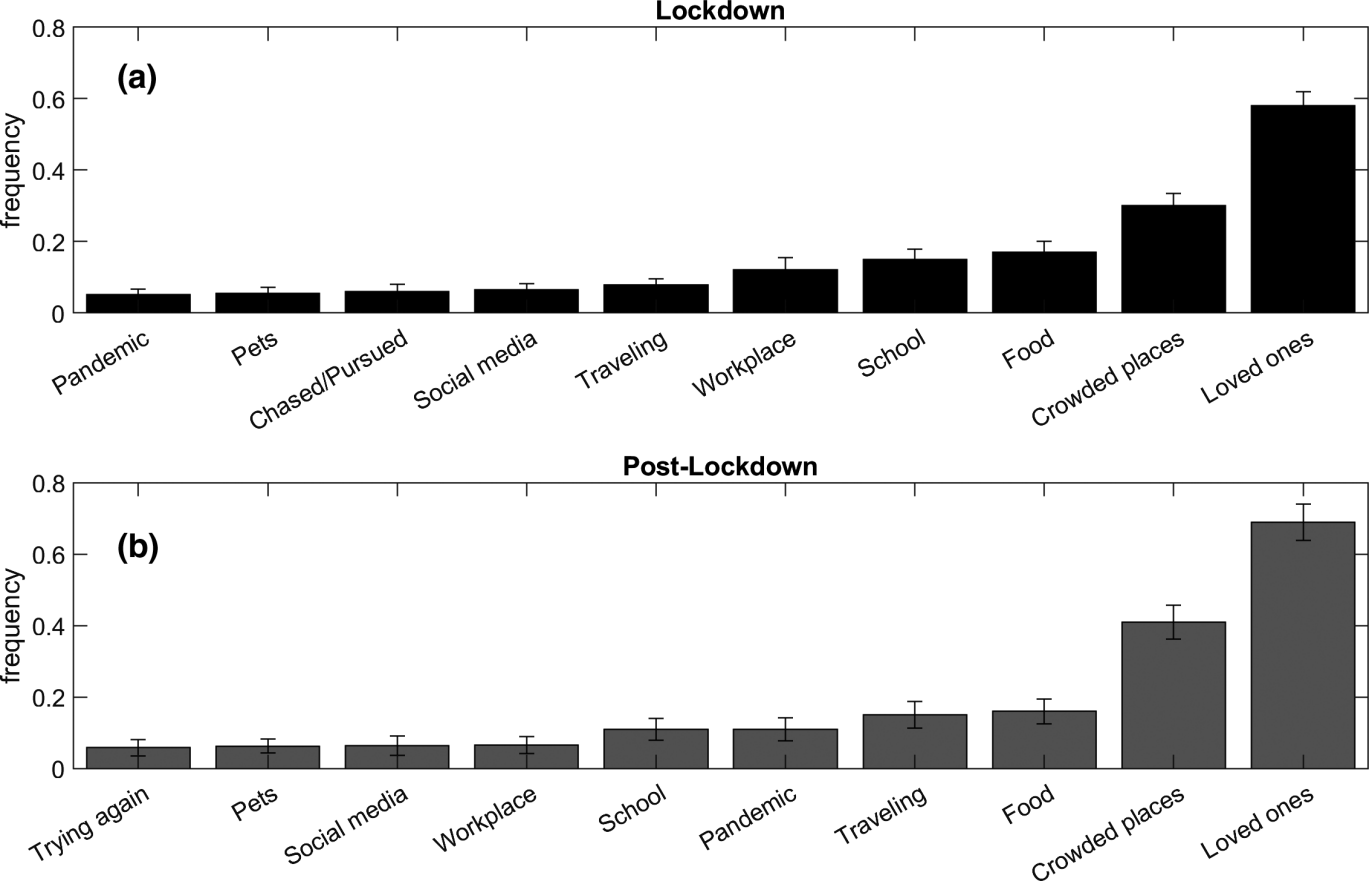
Frequency of dream contents. The bars represent the 10 most frequent dream contents during lockdown (a) and post‐lockdown (b). Error bars represent the standard errors

**FIGURE 3:**
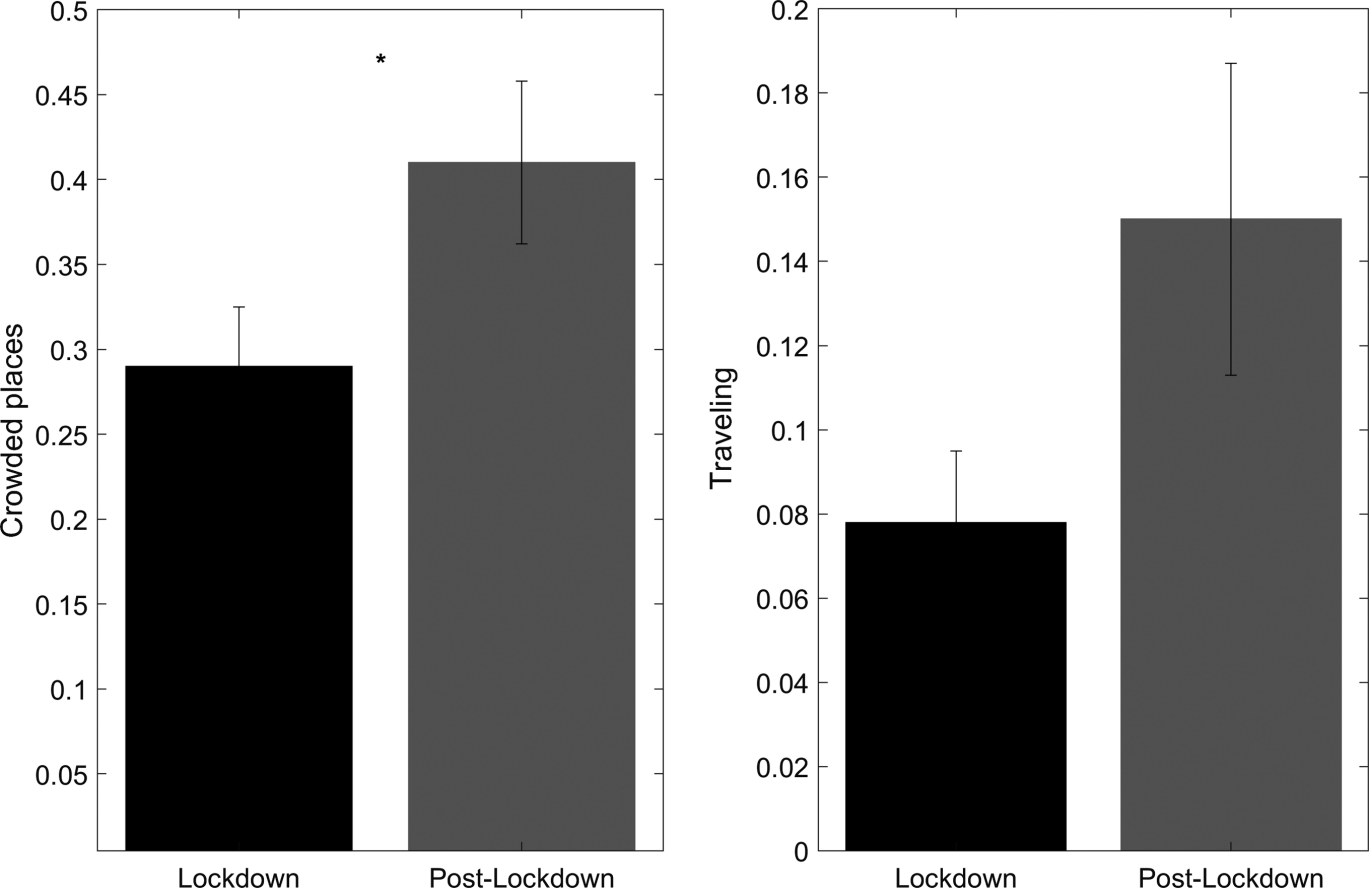
Dream contents: comparisons between lockdown and post‐lockdown period. Frequency of dream contents exhibiting a significant (crowded places: asterisked; *p* < 0.05) or almost significant (travelling: *p* = 0.05) difference (univariate repeated‐measures ANOVAs) between lockdown (black bars) and post‐lockdown (grey bars). Error bars represent the standard errors

Figure 2 depicts the 10 dream contents more frequently reported during lockdown and post‐lockdown. Loved ones, crowded places and eating were the most frequent dream contents in both weeks. Statistical comparisons showed that subjects had more dreams, including “being in crowded places”, during post‐lockdown than lockdown (*F*_1,58_ = 4.813, *p* = 0.032, *η* ^2^ = 0.077; Figure 3). Moreover, for illustrative purposes, we highlighted that “travelling” showed a trend in the same direction, namely, subjects had more dreams including travelling during post‐lockdown compared with lockdown (*F*_1,58_ = 3.91, *p* = 0.05, *η* ^2^ = 0.063; Figure 3).

## 4 DISCUSSION

To the best of our knowledge, the current study provided the first within‐subjects longitudinal assessment of dream activity across the pandemic in Italy. Our results from a within‐subjects design confirmed that both sleep and dream measures showed critical differences between the lockdown and post‐lockdown periods.

Regarding sleep measures, we revealed that the confinement period was characterized by a higher number of awakenings and greater perceived difficulty falling asleep than the subsequent week. These results are substantially coherent, with several findings reporting lower sleep quality during the Italian lockdown (Cellini et al., 2020). Consistently, self‐reported difficulties concerning sleep initiation and sleep maintenance were also reported in a recent Canadian study by Robillard et al. (2021) comparing outbreak with pre‐outbreak period.

It is easy to speculate that the greater difficulty to fall asleep and the sleep fragmentation across the night may be linked with the higher arousal due to the psychological distress experienced during wakefulness. Indeed, consistently with previous findings (Casagrande et al., 2020), our descriptive data revealed that more than half of our sample showed clinically relevant anxiety and PTSD‐related symptoms during sleep at T0 (lockdown period). We have to underline that our sample was composed of 80% women, who seem to be more affected by the pandemic, both concerning sleep quality and mental health (Salfi et al., 2020).

Crucially, we showed that subjects had higher dream recall and lucid dreams frequency during lockdown compared with the post‐lockdown week. On the one hand, this result is in line with our expectations. Consistently with recent cross‐sectional studies (Gorgoni et al., 2021; Iorio et al., 2020; Scarpelli, Alfonsi, Mangiaruga, et al., 2021; Schredl & Bulkeley, 2020), we confirmed with a longitudinal approach the greater dream production during confinement, a very stressful period due to the announcement of the pandemic and containment measures. The relationship between traumatic events and DRF replicates previous findings (Hartmann and Brezler, 2008; Tempesta et al., 2013). Indeed, more intense dream activity was observed immediately after the Twin Towers attack (Hartmann and Brezler, 2008). Similarly, survivors of the seismic disaster in central Italy (August 2009) showed higher nightmares in the area near the epicentre (Tempesta et al., 2013). In keeping with the idea that COVID‐19 represents a “collective trauma”, a recent study observed the reactivation of PTSD symptoms in patients in remission during lockdown (Gupta, 2020).

To the best of our knowledge, our result regarding the higher lucid dream frequency during lockdown compared with post‐lockdown represents a completely original finding. Lucid dreaming is a particular mental sleep activity in which individuals are aware of their dreams while remaining asleep (de Macêdo et al., 2019). This peculiar form of dreaming may provide an opportunity to increase self‐control and emotional regulation in individuals experiencing adverse events (Scarpelli et al., 2019). In these terms, the greater presence of lucid dreams during confinement could reflect the attempt to improve the coping ability in the waking state (de Macêdo et al., 2019). Some evidence revealed a beneficial effect of lucid dreaming in attenuating distress of nightmares and reducing their frequency (Rak et al., 2015).

It should be hypothesized that the higher number of awakenings and greater dream activity during lockdown may be linked. Intra‐sleep awakenings may promote the storage of oneiric materials and, consequently, the recall upon morning awakenings (Koulack & Goodenough, 1976; van Wyk et al., 2019). However, the two phenomena did not appear directly associated in our study, as no significant correlation was found (we tested the hypothesis performing Pearson's correlations). Hence, we suggest that the relationship between sleep measures and dreaming is more complex and requires further deepening. Actually, the current literature highlights that objective measures provided by polysomnography and quantitative electroencephalogram (EEG) analysis represent the better way to obtain additional and reliable information on sleep fragmentation and microstructural sleep variables affecting dream recall (Scarpelli, Alfonsi, Gorgoni, et al., 2021). In this direction, the electrophysiological (EEG) studies revealed that a greater desynchronized EEG (i.e. higher rapid frequencies and reduced slow oscillations) predicts dream recall (Scarpelli, Bartolacci, et al., 2020; Scarpelli, D'Atri, et al., 2020; Siclari et al., 2017).

Interestingly, we revealed that during post‐lockdown, people had more dream content regarding crowded places. This finding may be interpreted in light of the “continuity‐hypothesis” (for a review, see Scarpelli, Bartolacci, et al., 2020). Indeed, the ease of restrictive measures during the second week of our protocol allowed people to go out and start over a relatively ordinary routine, facing the external world for the first time after prolonged confinement. We suppose that the possibility to go out and access places frequented by other people could represent a significant experience for each individual after more than 50 days of isolation. According to the recent literature, personally significant events and concerns have been easily represented into a dream scenario (Eichenlaub et al., 2019). Conversely, no difference was observed for contents directly related to the pandemic/epidemic. Nevertheless, in keeping with other findings on pandemic dreams (Pesonen et al., 2020), it appeared to be one of the most frequent contents for the entire period of dream recording. Although restrictive measures were eased after May 4, the pandemic continued to influenced individuals' daily life also during post‐lockdown, therefore we cannot expect significant changes concerning this theme in oneiric contents.

Surprisingly, we found no difference in qualitative dream features. Considering the within‐subjects design, the lack of differences concerning qualitative characteristics could be ascribed to the fact that trait‐like features may stably influence dream report, and the post‐lockdown period is too close to T0 to appreciate large differences in terms of dream features concerning emotional intensity, visual vividness and bizarreness.

Finally, we have to mention some methodological limitations: (a) the short period of data collection: providing a longer data collection likely would allow us to detect larger differences, especially concerning emotional features of dreaming. At the same time, it should be considered that restricting the study to a 2‐week period was necessary so as to minimize fatigue effects in providing full reports of dreams. Indeed, longer schedules of collecting dream recall could lead to fatigue in participant compliance. In these terms, we cannot rule out the possibility of the post‐lockdown portion of data collection being made inaccurate due to this reason; (b) the low percentage of men and the limited age range (19–41 years) make the sample scarcely representative of the whole Italian population; (c) no objective sleep measures were collected, not allowing to ascertain information provided on sleep quality.

Undoubtedly, the main advantage of the present study is represented by applying a prospective method that allowed the reduction of mnestic biases and collection, for the first time, of reliable intra‐individual repeated measures during the pandemic.

## CONFLICT OF INTEREST

None of the authors has potential conflicts of interest to be disclosed.

## AUTHOR CONTRIBUTIONS

Serena Scarpelli: Conceptualization, Methodology, Validation, Formal analysis, Data curation, Supervision, Writing – original draft. Maurizio Gorgoni: Conceptualization, Methodology, Validation, Data curation, Supervision, Writing – original draft. Valentina Alfonsi: Investigation, Data curation. Ludovica Annarumma: Investigation, Data curation. Valentina di Natale: Investigation, Data curation. Emilio Pezza: Investigation, Data curation. Luigi De Gennaro: Conceptualization, Methodology, Validation, Supervision, Writing – original draft.

## Data Availability

The data that support the findings of this study are available from the corresponding author upon request.
